# Structural Stability, Electronic, Mechanical, Phonon, and Thermodynamic Properties of the M_2_GaC (M = Zr, Hf) MAX Phase: An ab Initio Calculation

**DOI:** 10.3390/ma13225148

**Published:** 2020-11-16

**Authors:** Muhammad Waqas Qureshi, Xinxin Ma, Guangze Tang, Ramesh Paudel

**Affiliations:** 1 State Key Laboratory of Advanced Welding and Joining, Harbin Institute of Technology, Harbin 150001, China; waqasmse@hit.edu.cn; 2 School of Material Science & Engineering, Harbin Institute of Technology, Harbin 150001, China; oaktang@hit.edu.cn; 3 Nepal Academy of Science and Technology (NAST), Khumaltar, Lalitpur 44700, Nepal; r.paudel@hit.edu.cn

**Keywords:** MAX phase, first-principles, M_2_GaC, mechanical properties, thermodynamic properties

## Abstract

The novel ternary carbides and nitrides, known as MAX phase materials with remarkable combined metallic and ceramic properties, offer various engineering and technological applications. Using ab initio calculations based on generalized gradient approximation (GGA), local density approximation (LDA), and the quasiharmonic Debye model; the electronic, structural, elastic, mechanical, and thermodynamic properties of the M_2_GaC (M = Zr, Hf) MAX phase were investigated. The optimized lattice parameters give the first reference to the upcoming theocratical and experimental studies, while the calculated elastic constants are in excellent agreement with the available data. Moreover, obtained elastic constants revealed that both the Zr_2_GaC and Hf_2_GaC MAX phases are brittle. The band structure and density of states analysis showed that these MAX phases are electrical conductors, having strong directional bonding between M-C (M = Zr, Hf) atoms due to M-d and C-p hybridization. Formation and cohesive energies, and phonon calculations showed that Zr_2_GaC and Hf_2_GaC MAX phases’ compounds are thermodynamically and dynamically stable and can be synthesized experimentally. Finally, the effect of temperature and pressure on volume, heat capacity, Debye temperature, Grüneisen parameter, and thermal expansion coefficient of M_2_GaC (M = Zr, Hf) are evaluated using the quasiharmonic Debye model from the nonequilibrium Gibbs function in the temperature and pressure range 0–1600 K and 0–50 GPa respectively.

## 1. Introduction

A new class of nanolayered transition metal carbides and/or nitrides was first discovered by H. Nowotny and coworkers, which were called H-phases in 1960, now known as MAX phases [1]. The general formula M_n+1_AX_n_ typically represents these ternary compounds (where M is an early transition metal, A is an IIIA- or IVA-group element, and X is either C or N, 1 ≤ n ≤ 3). MAX phase crystallizes in the hexagonal crystal structure (space group P6_3_/mmc) in which MX_6_ octahedral crafted with a pure A-group atom layer grown in c-direction, which bring forth a mixture of a strong covalent M-X bond and a relatively weak metallic M-A bond [2,3]. Due to a unique layered structure, MAX phases possess hybrid properties of both metals and ceramics, such as low density, machinability, thermal and electrical conductivity, damage and irradiation tolerance, oxidation, and corrosion resistance [4,5,6,7]. These remarkable properties make MAX phases suitable for versatile applications from microelectronics to aerospace [8,9,10,11]. Moreover, these ternary compounds can be exfoliated into 2D nanocrystals (MXene) by the selective etching of A-elements, applicable in Li-ion and sodium-ion batteries and supercapacitors, which further broaden the area of interest for researchers [12,13,14].

Over the past few years, extensive work has been devoted to investigating the properties of MAX phases materials experimentally and theoretically. More than 70 different thermodynamically stable M_n+1_AX_n_ materials have been synthesized experimentally in bulk form and some of them as a thin-film. Experimental results proved that Al-based MAX phase materials are thermally stable due to the formation of a continuous protective Al_2_O_3_ layer [15,16]. Recently, among the M_2_AC MAX phases, Zr_2_AlC and Hf_n+1_AlC_n_ (n = 1, 2) have already been synthesized, and their lattice parameter was in good agreement with first-principle investigations [17,18]. As far as theoretical research is concerned, density functional theory (DFT) [19] calculations have provided valuable information related to MAX phases. For M_2_GaC MAX phases, X.-X. Pu et al. [20] calculated the structural, electronic, and thermodynamic properties of Ti_2_GaC, Thore et al. [21] studied the electronic, elastic, and vibrational properties of Mn_2_GaC, and Shein et al. [22,23] investigated the structural, elastic, and electronic properties of Mo_2_GaC. At the same time, Qing-He et al. [24] found the extraordinary structural evolution while calculating the mechanical properties of Mo_2_GaC at different pressures. In addition, A. Petruhins et al. [25] predicted the phase stability, and the magnetic state of Cr_2_GaC and thin film of Cr_2_GaC was also prepared using the magnetron sputtering technique. In particular, Ga-containing M_n+1_GaC_n_ phases (where M = Ti, Cr, and n = 1, 3) were synthesized by J. Etzkorn et al. in 2009 [26].

Similarly, many computed data can be found in the literature for M_2_AC (M = Zr, Hf) MAX phases [27,28,29,30]. For example, A. Bouhemadou et al. investigated the structural, electronic, and elastic properties of a wide range of M_2_AC MAX phases [31], B. Ghebouli, et al. [32] computed the structural, elastic, and thermal properties of M_2_SiC, and Fen Luo et al. [33] studied the thermodynamic properties of Zr_2_AlC MAX phase under high pressure and temperature respectively. Despite numerous theoretical investigations, the computed data related to M_2_GaC (M = Zr and Hf) is lacking. To the authors’ best knowledge, only limited information about the elastic properties of M_2_GaC (M = Zr and Hf) has been reported by Sun Zhimei et al. [34]. Furthermore, thermodynamic properties of M_2_GaC MAX phases at higher temperature and pressure have not been studied yet. So, our results can serve as a reference for upcoming theoretical and experimental studies.

In the present study, density functional theory and the quasiharmonic Debye model were utilized to study the electronic, structural, and mechanical properties of M_2_GaC MAX phase materials along with their thermodynamic properties at pressure ranging 0–50 GPa and temperature ranging 0-1600 K. The Debye temperature $\left(\theta_{D}\right)$, heat capacity (C_v_), thermal expansion (α) coefficient, and Grüneisen parameter (γ) were calculated in the described pressure and temperature ranges. This article is organized as follows: there are detailed computational methods in Section 2; the obtained results and discussion of M_2_GaC are presented in Section 3; in Section 4, thermodynamic properties are discussed; and in Section 5, a summary of this research is given.

## 2. Computational Details

The ab initio estimation was performed based on density functional theory (DFT) using the Cambridge Serial Total Energy Package (CASTEP) [35] in which plane wave ultrasoft pseudopotential is used. The exchange-correlation was treated within the generalized gradient approximation (GGA) of Perdew–Wang (GGA-PW91) [36], Perdew–Burke–Ernzerhof (PBE) [37], and local density approximations developed by Ceperly and Alder and parametrized by Perdew and Zunger (LDA-CA-PZ) [38] for comparison. GGA-PBE was implemented to calculate the different properties in each system. A total energy convergence test was performed carefully at additional cutoff energies and the k-point mesh for the M_2_GaC (M = Zr and Hf) system. For all cases, the plane waves’ cut off energy, and the Monkhorst-pack [39] scheme k-point were set at 600 eV and 15 × 15 × 3, respectively. The ground state structural parameters were determined using the Broyden–Fletcher–Goldfarb–Shanno (BFGS) [40] minimization technique while the self-consistent convergence parameters were total energy tolerance less than 5 × 10^−6^ eV/atom, stress component less than 0.02GPa, maximum force tolerance 0.01eV/ Å, and maximum displacement of atom during the geometric optimization less than 0.0005 Å. To investigate the thermodynamic properties of Zr_2_GaC and Hf_2_GaC, the quasiharmonic Debye model using the GIBBS program [41,42] was applied in the temperature ranges 0–1600 K and pressure in the range 0–50 GPa. Finally, the phonon dispersion was computed using a finite displacement method implemented in Material Studio [43].

## 3. Results and Discussion

### 3.1. Structural Properties

**Figure 1:**
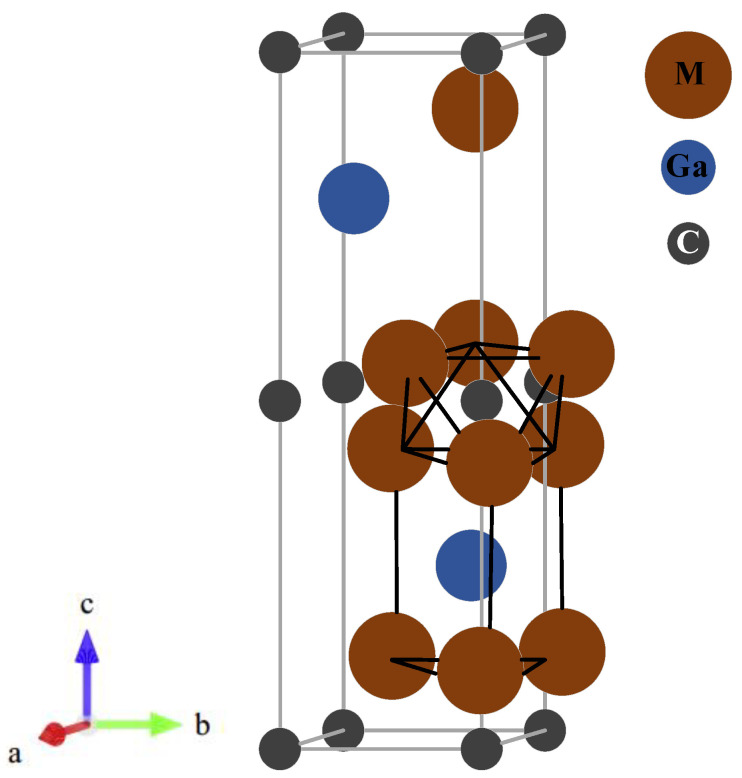
The unit cell of the M_2_GaC MAX phase (M = Zr, Hf). An edge-shared [M_6_X] octahedra, and a M_6_A trigonal prism are outlined.

**Figure 2:**
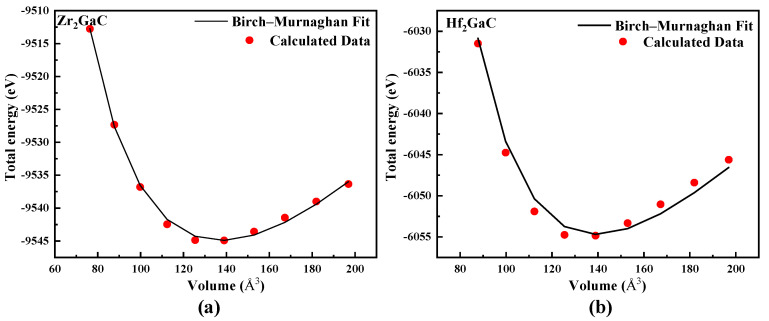
The total energy as a function of unit cell volume for the M_2_GaC MAX phase (**a**) Zr_2_GaC; (**b**) Hf_2_GaC.

As mentioned earlier, MAX phase materials crystallize in hexagonal crystal structures with space group P6_3_/mmc (No. 194) in which an edge-shared octahedral of transition metal carbide M_6_X is sandwiched between a pure A-element. The stimulated unit cell is shown in Figure 1. The Wyckoff positions in the M_2_GaC (M = Zr and Hf) system are as follows: C 2a(0, 0, 0), Ga 2d(2/3, 1/3, 1/4), and M 4f(1/3, 2/3, *Z_M_*) where the internal parameter *Z_M_* is about 0.08. The quest for stable structure and optimized geometry of the crystalline structure is the first step in any ab initio calculation. For this purpose, the total energy versus volume for each structure in the M_2_GaC (M = Zr and Hf) system is plotted in Figure 2, and the data were fitted according to the equation of energy of state (EOS) due to Birch–Murnghan [44]. For comparison, we have obtained lattice parameters for the M_2_GaC MAX phase by GGA-PW91, LDA-CA-PZ, along with GGA-PBE functionals, and the results are shown in Table 1. Moreover, there is no experimental data available in the literature related to M_2_GaC (M = Zr and Hf), so as a reference, theoretical and experimental results of other Ga-containing MAX phases, i.e., Ti_2_GaC, Cr_2_Gac, and Sc_2_GaC by other researchers are also cited in Table 1 for comparison.

The computed equilibrium volume for Zr_2_GaC and Hf_2_GaC is 136.92 ${Å}^{3}$ and 134.20 ${Å}^{3}$, and lattice parameters (a, c) are 3.33 Å, 14.25 Å for Zr_2_GaC, and 3.32 Å, 14.02 Å for Hf_2_GaC, respectively. The study made by Sun Zhimei et al. [34] on Ga-containing MAX Phases M_2_GaC (where M = Ti, Zr, Hf, V, Nb, Ta, Cr, Mo, W) did not contain useful information related to optimized lattice parameters or volume, and there is no experimental data available in the literature so far. Hence, we predict the physical properties of the M_2_GaC MAX phase for the very first time.

**Figure 3:**
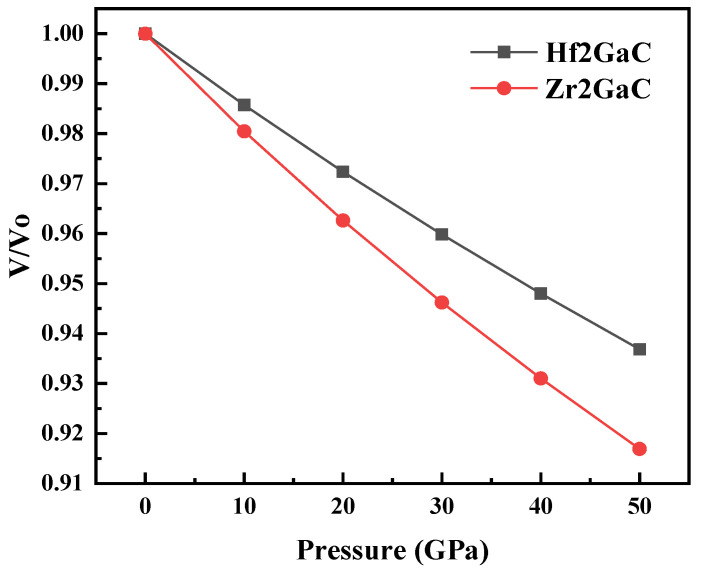
Calculated dependence of volume change *V/V_0_* on pressure for M_2_GaC MAX phase (M = Zr, Hf).

The effect of pressure on the equilibrium volume of M_2_GaC, ranging from 0–50 GPa with a step of 10 GPa, is shown in Figure 3, where *V*_0_ is the volume at the zero-pressure equilibrium structural parameter. It is noticed that the volume ratio (*V*/*V*_0_) was reduced in the order of ${\left(V/V_{0}\right)}_{Zr_{2}GaC}=9\%>{\left(V/V_{0}\right)}_{Hf_{2}GaC}=7\%$ with an increase in pressure. Therefore, the compressibility of the M_2_GaC MAX phase system is strong, and the change of external pressure has a more significant impact on Zr_2_GaC.

The energy of formation per atom ($E_{for}^{M_{2}GaC}$) was calculated to investigate the phase stability of the researched M_2_GaC MAX phase, which is defined as follows [46]:

(1) $$E_{for}^{M_{2}GaC}=\frac{E_{total}^{M_{2}GaC}-\left(xE_{solid}^{M}+yE_{solid}^{Ga}+zE_{solid}^{C}\right)}{x+y+z}M=Zr,Hf$$

where: *x*, *y*, *z* is the number of atoms for *M*, *Ga*, and *C* element in the unit cell, i.e., *x* = 4, *y* = 2, and *z* = 2 and $E_{total}^{M_{2}GaC}$, $E_{solid}^{M}$, $E_{solid}^{Ga}$, and $E_{solid}^{C}$ are the total energy of M_2_GaC MAX phase, *M*, *Ga*, and *C* atoms in the solid form respectively in their stable structures. The calculated formation energy for Zr_2_GaC and Hf_2_GaC is −7.59 eV/atom and −7.45 eV/atom, respectively, given in Table 1.

Cohesive energy ($E_{Coh}$) is defined as the energy required to break the crystal into an isolated atom and used to identify the structural stability of the M_2_GaC MAX phases. The $E_{Coh}$ is computed using Equation (2).

(2) $$E_{Coh}^{M_{2}GaC}=\frac{E_{total}^{M_{2}GaC}-\left(2E_{Iso}^{M}+E_{Iso}^{Ga}+E_{Iso}^{C}\right)}{4}M=Zr,Hf$$

where: $E_{Coh}^{M_{2}GaC}$, $E_{Iso}^{M}$, $E_{Iso}^{Ga}$, and $E_{Iso}^{C}$ are the total energy of the M_2_GaC MAX phase and the energies of isolated M, Ga, and C atoms, respectively. The calculated $E_{Coh}$ for Zr_2_GaC and Hf_2_GaC is −6.328 eV/atom and −6.162 eV/atom, respectively (see Table 1). It is worth noticing that the formation and cohesive energies for M_2_GaC are negative, indicating that these MAX phases are energetically favorable from the thermodynamic point of view. Moreover, these structures can be experimentally formed by different synthesis methods.

### 3.2. Electronic Properties

#### 3.2.1. Band Structure

**Figure 4:**
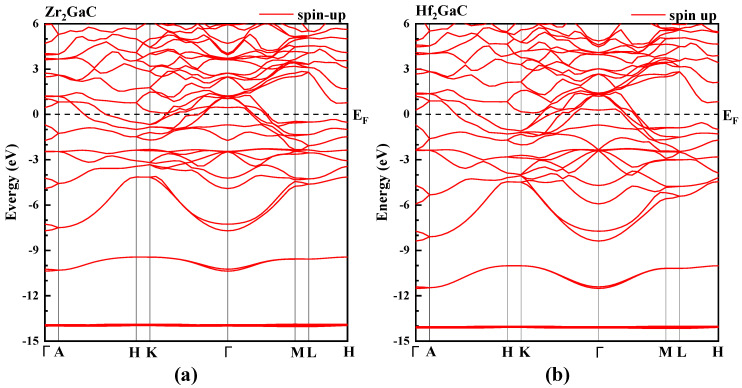
The calculated band structure as obtained from GGA-PBE of M_2_GaC MAX phase (**a**) Zr_2_GaC; (**b**) Hf_2_GaC.

Based on optimized lattice parameters, the band structure for M_2_GaC (M = Zr, Hf) was calculated and obtained. Figure 4 shows the band structure from −15 eV to 6 eV energy range along the high symmetry lines of the Brillouin zone. The appearance of the obtained band structure for both Zr_2_GaC and Hf_2_GaC MAX phases is similar to the other metallic MAX phases, such as Cr_2_AlC [47] and Ti_2_AlC [48] because of considerable overlapping of bands without having bandgap in the vicinity of the Fermi level. It can also be assessed that there is a strong anisotropic behavior with less c-axis energy dispersion. In other words, less energy dispersion was observed along the short H-K and M-L directions for both MAX phase compounds indicating the electronic anisotropic nature of M_2_GaC MAX phases (see Figure 4), which means conductivity will be lower along the c-axis as compared to their basal planes. The electronic anisotropic behavior of M_2_GaC MAX phases is in good agreement with the available data of other MAX phases [21,49]. This is a consequence of the nanolaminated structure of M_2_GaC MAX phases. Vincent et al. [50] investigated the electronic anisotropy of Ti_2_AlC MAX phase and found that resistivity (*ρ = 1/σ*) along the c-axis is much higher than polycrystalline bulk material or thin-film along the (0001) orientation.

#### 3.2.2. Density of States (DOS)

**Figure 5:**
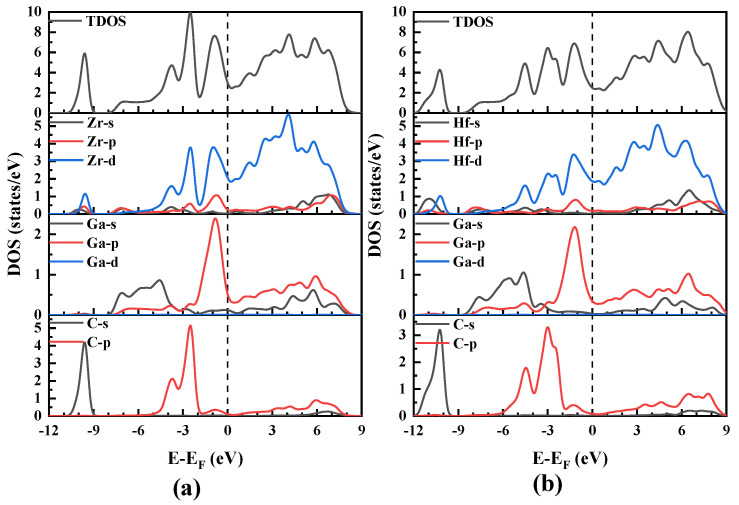
The total and partial density of states (PDOS) obtained from GGA-PBE for M_2_GaC MAX phase). Fermi level is set to 0 eV. (**a**) Zr_2_GaC; (**b**) Hf_2_GaC.

**Figure 6:**
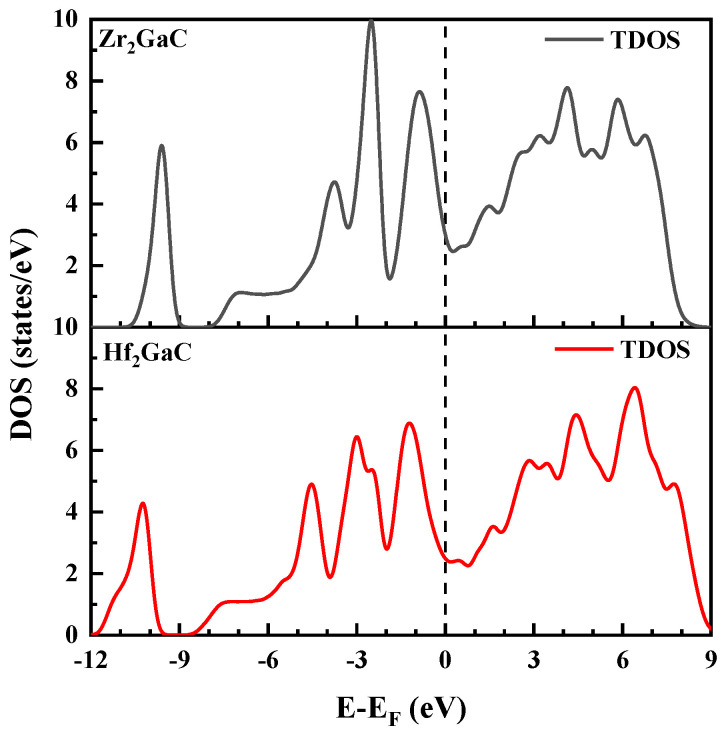
The total density of states obtained from GGA-PBE for M_2_GaC MAX phase (M = Zr, Hf). Fermi level is set to 0 eV.

To investigate the nature of chemical bonding in the M_2_GaC (M = Zr, Hf) MAX phases, the density of states (DOS) was calculated and studied. The total and partial density of states is shown in Figure 5 and Figure 6 and the results obtained are tabulated in Table 1. It is observed that the total DOS at E_F_ for Zr_2_GaC and Hf_2_GaC are 3.002 states/eV/unit cell and 2.475 states/eV/unit cell, respectively, suggesting that the both Zr_2_GaC and Hf_2_GaC MAX phases are metallic and the metallicity of M_2_GaC MAX phase compounds can be measured by DOS at E_F._ The metallicity of Zr_2_GaC and Hf_2_GaC phases at ambient temperature is investigated by:

(3) $$f_{m}=\frac{n_{m}}{n_{e}}=\frac{k_{B}T\times N\left(E_{F}\right)}{n_{e}}=\frac{0.026\times N\left(E_{F}\right)}{n_{e}}$$

where *n_m_* and *n_e_* are the thermally excited number of electrons and the total number of valence electron in the unit cell while *k_B_*, *T* and *N(E_F_)* are the Boltzmann constant, temperature, and value of DOS at *E_F_* in unit states/eV/unit cell, respectively. The calculated values of *f_m_* are listed in Table 1. Moreover, the conductivity of the M_2_GaC MAX phase is investigated from the DOS at *E_F_* as follows:

(4) $$v_{F}=\sqrt{\frac{2E_{F}}{m}}$$

where $v_{F}$ and m are the velocity of electrons near the Fermi level and mass of electrons, respectively. From $v_{F}$, the conductivity (*σ*) of material is estimated as:

(5) $$\sigma =\frac{ne^{2}\tau }{m}=\frac{ne^{2}l}{mv_{F}},\tau =\frac{l}{v_{F}}$$

where: *n*, *e*, and *l* are the number of electrons, electrons’ charge, and mean free path of electrons, respectively. τ is the time between two collisions. In Equation (5), *e*, *l*, and *m* are constants; thus, conductivity mainly depends upon the $n/v_{F}$ ratio. It is observed that the ${\left(n/v_{F}\right)}_{Zr_{2}GaC}>{\left(n/v_{F}\right)}_{Hf_{2}GaC}$; we may conclude that the conductivity of the M_2_GaC MAX phase is in the order of Zr_2_GaC > Hf_2_GaC. However, the values for total density of states (TDOS) obtained for M_2_GaC (M = Zr, Hf) is much smaller than that of Cr_2_AlC (6.46 states/eV/unit cell) [43], the maximum TDOS measured among the MAX phases so far. It is noted that the main contribution in the DOS at E_F_ is from the M-4d electrons in both cases, indicating that d bands of transition metal mainly contribute to the conduction properties during the electrical transport. These results are consistent with the previous studies on MAX phases [46].

For Zr_2_GaC, the total and partial density of states (TDOS and PDOS) from Figure 5a shows that the lowest states range from −10.85 eV to −9.11 eV of the TDOS is formed by C-s with Zr-d, Zr-p along with a small portion of Zr-s. The higher states from −8.01 eV to −5.1 eV are entirely formed by Ga-s states. The valance band in the range −5 eV to −1.90 eV is formed by the strong hybridization of C-s and Zr-d states. The highest valance band is related to the relatively weak hybridization of Zr-d, Zr-p, and Ga-p, which shows the covalent interaction between the Zr-d and Ga-p. The TDOS and PDOS for Hf_2_GaC shown in Figure 5b is similar to that of Zr_2_GaC with two common sharing features: Firstly, as M from Hf to Zr, the hybridization peaks of C-s and C-p with M-d states shifts from right to left towards the lower energy level and C-s, C-p peaks become narrow (See Figure 6). This expresses that the weakened M-d and C-s, C-p covalent interaction, results in decreased bulk modulus for the M_2_GaC MAX phase, as given in Table 1. The indicated peak shift is seen in the M_2_GaC MAX phase TDOS in Figure 6. Secondly, the M-d (M = Zr, Hf) and C-p hybridization peak lies between −5 eV and −1.90 eV, while M-d and Ga-p is located between −1.90 eV and 0 eV. This indicates that bonding between the M-d and C-p states is stronger than that of the M-d and Ga-p states.

### 3.3. Mechanical Properties

A brief knowledge about the elastic constants of the crystalline materials helps to predict its behavior under the application of external stress. It contributes to a critical understanding of the many solid-state properties, i.e., ductility, brittleness, stiffness, structural stability, and anisotropy. The elastic constants (C_ij_) of M_2_GaC (M = Zr, Hf) were calculated from the PBE, PW91, and LDA, and the obtained results are tabulated in Table 2. The hexagonal structure of MAX phases has six independent elastic constants (C_11_, C_12_, C_13_, C_33_, C_44_=C_55,_ and C_66_), but five of them are listed since C_66_ = $\frac{\left(C11-\;C12\right)}{2}$ [51]. Moreover, it is observed that the obtained elastic constants (C_ij_) are positive and satisfy the mechanical stability criteria known as Born stability (C_11_ > 0, C_11_−C_12_ > 0, C_44_ > 0, C_66_ > 0, (C_11_ + C_12_) C_33_−2$C_{13}^{2}$ > 0) [52] showing that the M_2_GaC MAX phase is mechanically stable. In order to comprehend the mechanical properties further, the bulk modulus (B), shear modulus (G), Young’s modulus (E), G/B or B/G (Pugh ratio), elastic anisotropy (A), and Poisson’s ratio (ν) are calculated from the obtained elastic constants and results are given in Table 3. The Voigt (V) [53], Russ (R) [54,55], and Voigt–Russ and Hill (VRH) [56,57] approximation scheme were used to determine the parameters concerning properties. The following equations are used to calculate these quantities:

(6) $$B=\frac{1}{2}\left(B_{V}+B_{R}\right)$$

and

(7) $$G=\frac{1}{2}\left(G_{V}+G_{R}\right)$$

where *Bv*, *Gv* and *Br*, *Gr* are the *B* and *G* in terms of the Voigt and Russ approximation respectively and calculated by given Equations (8)–(11)

(8) $$B_{V}=\frac{1}{9}\left(2\left(C_{11}+C_{12}\right)+4C_{13}+C_{33}\right)$$

(9) $$G_{V}=\frac{1}{30}\left(C_{11}+C_{12}+2C_{33}-4C_{13}+12C_{44}+12C_{66}\right)$$

and

(10) $$B_{R}=\frac{\left(\left(C_{11}+C_{12}\right)C_{33}-2C_{12}^{2}\right)}{\left(C_{11}+C_{12}+2C_{33}-4C_{13}\right)}$$

(11) $$G_{R}=\frac{\frac{5}{2}\left[{\left(\left(C_{11}+C_{12}\right)C_{33}-2C_{12}^{2}\right)}^{2}\right]C_{55}C_{66}}{\left[3B_{V}C_{55}C_{66}+{\left(\left(C_{11}+C_{12}\right)C_{33}-2C_{12}^{2}\right)}^{2}\left(C_{55}+C_{66}\right)\right]}$$

Young’s modulus can be calculated by

(12) $$E=\frac{9BG}{3B+G}$$

To calculate the anisotropy index (*A*) following expression is used

(13) $$A=\frac{4C_{44}}{C_{11}+C_{33}-2C_{13}}$$

The Poisson’s ratio can be calculated by:

(14) $$\sigma =\frac{3B-2G}{2\left(3B+G\right)}$$

Generally, the calculated parameters B, G, and E measure the material’s resistance to fracture, resistance to plastic deformation, and stiffness of the material, respectively. The bulk modulus (B) calculated in terms of elastic constants is in good agreement with the bulk modulus (B*) obtained from the Birch–Murnghan equation of state (EOS), indicating that our estimated elastic constants for Zr_2_GaC and Hf_2_GaC are accurate and precise. Moreover, the calculated bulk modulus obtained from LDA is in good agreement with the available study [34]. Furthermore, B, G, and E for Hf_2_GaC > Zr_2_GaC, which means the effect to resist the deformation of Hf_2_GaC is better than that of Zr_2_GaC. Therefore, the reduction of volume ratio (*V*/*V*_0_) for Zr_2_GaC is higher than Hf_2_GaC (See Figure 3).

Another parameter, the Pugh’s ratio (B/G and G/B) [58], separates the ductile to brittle nature of the material. It is known that if B/G > 1.75 and G/B < 0.5, the material will be ductile; otherwise, it will be brittle [59]. In our cases (Zr_2_GaC and Hf_2_GaC), the B/G < 1.75 and G/B > 0.5; consequently, the compounds under this study are predicted to be brittle like Ta_2_GaC [20]. The anisotropy index (A) gives the knowledge about the anisotropic nature of the materials. If the value of A is equal to 1, then the material is said to be isotropic; otherwise, material will be anisotropic if the value of A is higher or lower than 1. Our calculated results from Table 3 showed that the M_2_GaC MAX phases are anisotropic.

One of the most essential elastic parameters is Poisson’s ratio (*σ*), defined as the ratio between the transverse strain to longitudinal strain under the applied tensile stress. It gives knowledge about the material’s chemical bonding and is linked to its stability against the shear stress. Whether the material is brittle or ductile can be predicted from the Poisson’s ratio (*σ*) value, and the 0.33 [60] value is set for ductile material; otherwise, the material is called brittle if *σ* < 0.33. In the results of both investigated Zr_2_GaC and Hf_2_GaC MAX phases, the Poisson’s ratio (*σ*) value is smaller; consequently, both compounds are predicted to be brittle.

**Figure 7:**
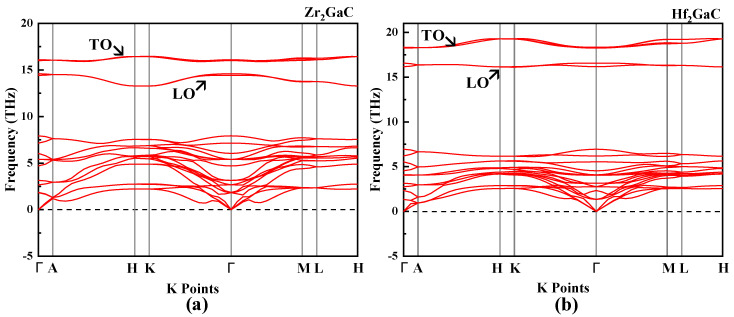
The phonon dispersion curve for M_2_GaC MAX phase (**a**) Zr_2_GaC; (**b**) Hf_2_GaC.

For the phase stability of M_2_GaC MAX phases, the calculated phonon dispersion curves along the high-symmetry directions in the Brillouin zone are shown in Figure 7. The M_2_GaC MAX phases have eight atoms in the unit cell; therefore, the phonon dispersion curve shows twenty-four branches (three acoustic and twenty-one optical). The optical frequencies (longitudinal optical (LO) and transverse optical (TO)) at Γ are 14.58 THz and 16.00 THz for Zr_2_GaC and 16.56 THz and 18.32 THz for Hf_2_GaC. We have not observed any negative or imaginary frequency in the phonon dispersion curves of both MAX phases indicating that M_2_GaC MAX phases are dynamically stable. To the best of the authors’ knowledge, phonon dispersion for Zr_2_GaC and Hf_2_GaC have not yet been investigated theoretically and experimentally.

## 4. Thermodynamic Properties

The thermal properties of Zr_2_GaC and Hf_2_GaC are computed in the temperature ranges 0–1600 K and the pressure in the range 0–50 GPa with the step of 10 GPa, whereas the quasiharmonic Debye model remains valid [41,42] in this temperature range. This model has been successfully applied to calculate the thermodynamic properties of other MAX phases as well. The Debye temperature (θ_D_) is calculated from elastic constants and it depends on the mean propagation sound velocity (*V_m_*). A couple of equations are used to calculate the θ_D_ which are given below: [61,62,63]

(15) $$\theta_{D}=\frac{h}{k_{B}}{\left[\frac{3n}{4\pi V_{a}}\right]}^{\frac{1}{3}}v_{m}$$

where

(16) vm=[13(2vt3+1v13)]−13

with

(17) $$v_{t}={\left(\frac{G}{\rho }\right)}^{\frac{1}{2}}$$

and

(18) $$v_{1}={\left(\frac{3B+4G}{3\rho }\right)}^{\frac{1}{2}}$$

where *h* is Planck’s constant, *k_B_* is Boltzmann’s constant, *n* represents the number of atoms per unit cell, *V_a_* is the atomic volume while *v_m_*, *v_t_*, and *v_l_* are mean sound velocity, transverse and longitudinal sound velocities respectively.

**Figure 8:**
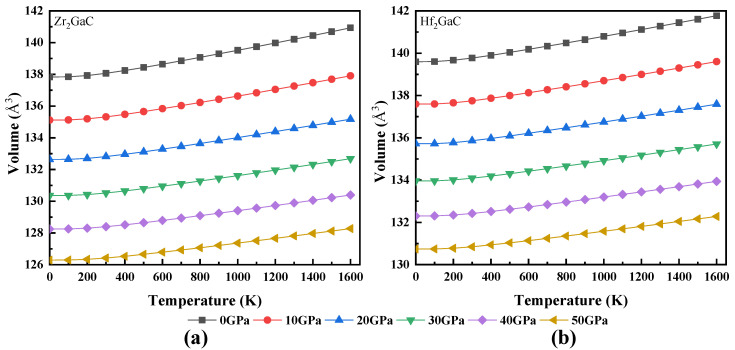
Temperature effect on lattice parameters at different pressures for M_2_GaC MAX phase (**a**) Zr_2_GaC; (**b**) Hf_2_GaC.

**Figure 9:**
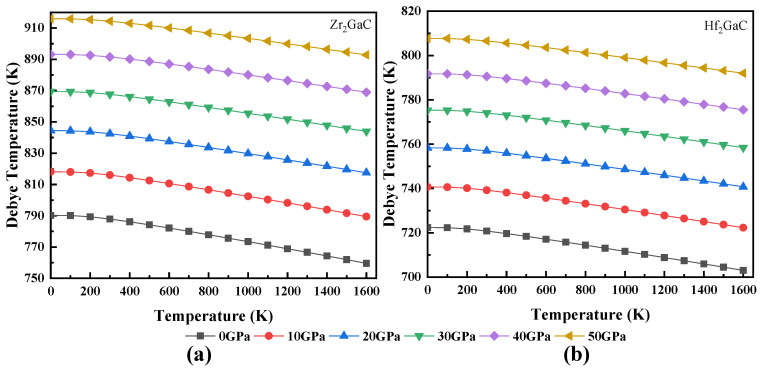
The dependence of Debye temperature on temperature at different pressures for M_2_GaC MAX phase (**a**) Zr_2_GaC; (**b**) Hf_2_GaC.

**Figure 10:**
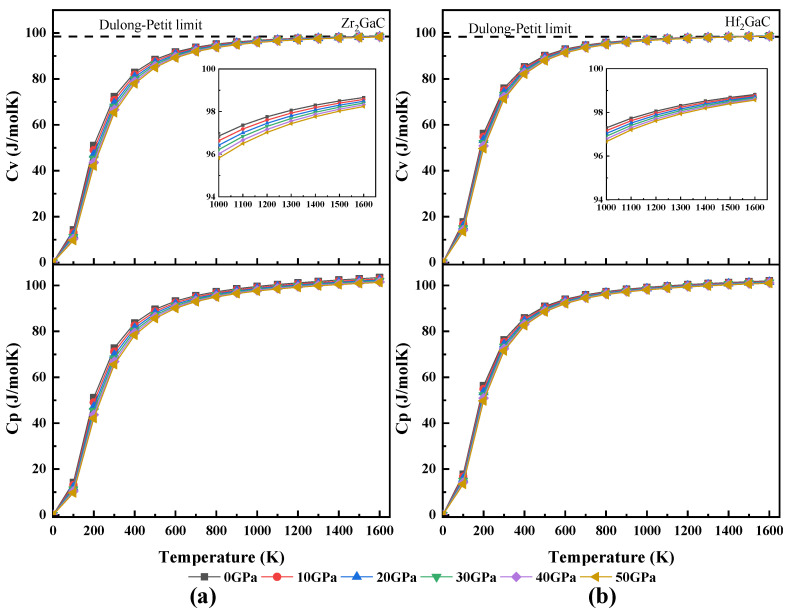
Temperature effect on heat capacity Cv and Cp at different pressures for M_2_GaC MAX phase (**a**) Zr_2_GaC; (**b**) Hf_2_GaC.

**Figure 11:**
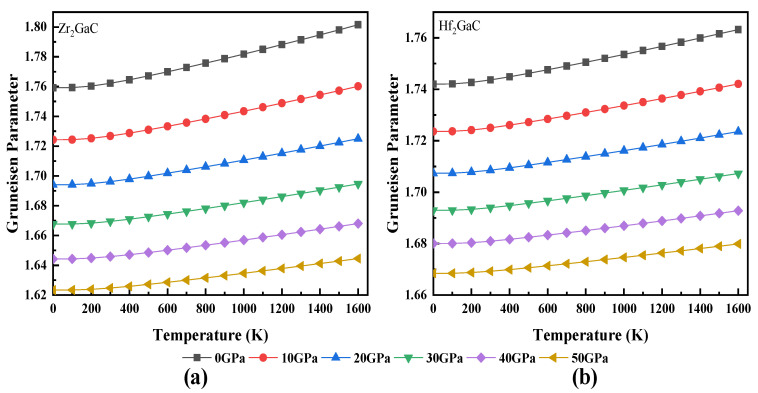
Temperature effect on Grüneisen parameters at different pressures for M_2_GaC MAX phase (**a**) Zr_2_GaC; (**b**) Hf_2_GaC.

**Figure 12:**
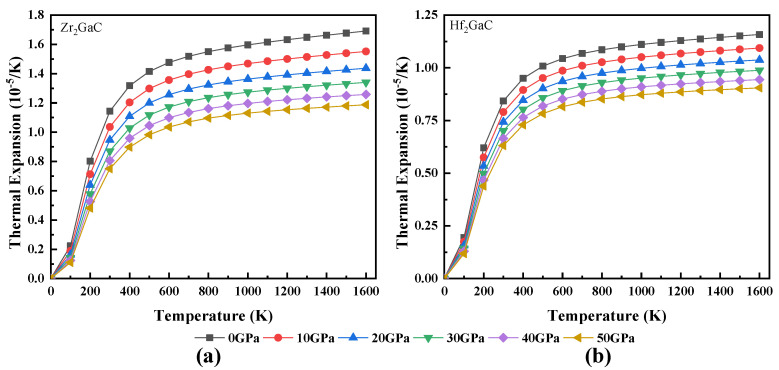
The effect of temperature on thermal expansion coefficient at different pressures for M_2_GaC MAX phase (**a**) Zr_2_GaC; (**b**) Hf_2_GaC.

The calculated relationship between volume and temperature, heat capacity and temperature, Debye temperature and temperature, Grüneisen parameter (γ) and temperature, and thermal expansion coefficient and temperature for Zr_2_GaC and Hf_2_GaC is given in Figure 8, Figure 9, Figure 10, Figure 11 and Figure 12. The calculated *v_m_, v_t_*, *v_l_,* Debye temperature (θ_D_), thermal expansion coefficient CTE (α), and heat capacity at constant volume (C_V_) and constant pressure (C_P_) for Zr_2_GaC and Hf_2_GaC at 300 K are presented in Table 1, Table 2, Table 3 and Table 4.

From Figure 8, it is noticed that a small change in volume takes place from the temperature 0 to 100 K at a given pressure, and volume linearly increases with the rise in temperature while a decrease in volume is observed at high pressure at a given temperature. The relationship between the Debye temperature and temperature is shown in Figure 9. The linear decline in Debye temperature could be seen with the increase in temperature and increases with pressure. For both Zr_2_GaC and Hf_2_GaC MAX phase, the value of *θ_D_* at a given temperature and pressure is in the order of ${\left(\theta_{D}\right)}_{Zr_{2}GaC}>{\left(\theta_{D}\right)}_{Hf_{2}GaC}$. Many physical properties of solidity such as hardness, thermal expansion [60], and heat capacity depend on Debye temperature. The hardness of the Zr_2_GaC and Hf_2_GaC MAX phase in terms of Debye temperature is in the order of Zr_2_GaC > Hf_2_GaC because the $\theta_{D}$ value for Zr_2_GaC and Hf_2_GaC is 787.91 K and 720.78 K, respectively, at 0 GPa and ambient temperature.

The effect of temperature on heat capacity (Cv, Cp) of Zr_2_GaC and Hf_2_GaC MAX phase is depicted in Figure 10. The heat capacity is the ability of the material to absorb heat from the surroundings, and one can get useful information about the density of states, lattice vibration, energy band structure, etc., from heat capacity. It is observed that both Cv and Cp increase abruptly with temperature when temperature limit T ≤ 300 K, beyond the 300 K, Cv and Cp increases slowly and finally get converged at higher temperature to obey the Dulong–Petit limit. The effect of temperature and pressure on heat capacity is the opposite; however, temperature change has a more significant impact than pressure. The obtained heat capacity values of Zr_2_GaC and Hf_2_GaC MAX phase at constant volume corresponding to ambient temperature and 0 GPa are 72.33 J/mol·K and 76.00 J/mol·K, respectively.

The Grüneisen parameter (γ) is used to calculate the thermal state and is a dimensionless quantity. The trend for γ is almost similar to that of volume. As shown in Figure 11 that γ decreases with an increase in pressure while increasing linearly with temperature. However, γ remains constant in the temperature range from 0–100 K. The obtained γ_0_ values for Zr_2_GaC and Hf_2_GaC are 1.76 and 1.74 at 0 GPa, respectively. Finally, the dependence of the thermal expansion coefficient (α) on temperature is depicted in Figure 12. At a given temperature, α decreases with the increase in pressure; this may be due to reduced unit cell volume. The results of α at ambient temperature and 0 GPa for Zr_2_GaC and Hf_2_GaC are 1.14 × 10^−5^ K^−1^ and 0.84 × 10^−5^ K^−1,^ respectively. However, in the range 0–300 K, α increases rapidly and reaches a plateau at higher temperature, indicating that the temperature effect is more important at lower temperature.

## 5. Conclusions

In this study, electronic, structural, elastic, phonon, thermodynamical, and mechanical properties of the M_2_GaC MAX phase (M = Zr, Hf) were comprehensively investigated by employing first-principle calculations based on GGA and LDA exchange-correlation functional. The thermodynamic properties were computed using the quasiharmonic Debye model in the pressure ranges 0–50 GPa, and temperature in the range 0–1600 K. The results showed that the formation and cohesive energies for both Zr_2_GaC and Hf_2_GaC are found to be negative, i.e., −7.59 eV/atom and −6.32 eV/atom for Zr_2_GaC and −7.45 eV/atom and −6.16 eV/atom for Hf_2_GaC. The PDOS revealed that the main contribution in electric transport at E_F_ was by transition metal M-4d electrons and hybridizations between M-d and C-p states is stronger than M-d and Ga-p states. The DOS at E_F_ for Zr_2_GaC and Hf_2_GaC was 2.96 states/eV/unit and 2.47 states/eV/unit, respectively, indicating that these MAX phases are predicted to be electrical conductors while band structures pointed out their anisotropic nature. Moreover, the formation and cohesive energies, elastic constants, and phonon calculations showed that both compounds are thermodynamically, mechanically, and dynamically stable. Furthermore, the values of E, B, and G in terms of elastic constants are in the order of Hf_2_GaC > Zr_2_GaC. From the B/G, Poisson’s ratio, and anisotropy index, it is concluded that the MAX phases investigated in this study are brittle and anisotropic. As far as thermodynamic properties of the M_2_GaC MAX phase are concerned, the volume and Grüneisen parameter increases linearly with an increase in temperature and decreases with an increase in pressure while the Debye temperature has an inverse trend compared to them. The heat capacities (Cv and Cp) and the thermal expansion coefficient increases rapidly up to the temperature ranges 0–300 K and then reaches a plateau at higher temperature. To the best of the authors’ knowledge, no data related to the electronic, structural, phonon, and thermodynamic properties in the literature exist so far. Hence, the results about the M_2_GaC (M = Zr, Hf) MAX phase can serve as a reference for future theoretical and experimental research.

## Figures and Tables

**Table 1:** Calculated lattice parameters (a) and (c) in Å, c/a, bulk modulus (GPa), no. of density of states (DOS)at E_F_ (states/eV/unit cell), and formation energy E_for_ (eV/atom) for M_2_GaC MAX phase (M = Zr, Hf) obtained by generalized gradient approximation (GGA) and local density approximation (LDA).

M_2_GaC	Functional	a (Å)	c (Å)	v (Å^3^)	c/a (Å)	B* (GPa)	N(E_F_) (states/eV/u.c)	E_for_ (eV/atom)	E_coh_ (eV/atom)	*f_m_* (×10^−3^)	Ref
Zr_2_GaC	GGA-PBE	3.330	14.257	136.92	4.28	121.8	3.002	−7.59	−6.328	5.20	This work
	GGA-PW91	3.330	14.245	136.88	4.27						
	LDA-CA-PZ	3.276	13.956	129.73	4.26						
Hf_2_GaC	GGA-PBE	3.324	14.025	134.20	4.22	141.9	2.475	−7.45	−6.162	4.29	This work
	GGA-PW91	3.325	14.003	134.11	4.21						
	LDA-CA-PZ	3.268	13.716	126.93	4.19						
Ti_2_GaC	GGA-PBE	3.083	13.397		4.34						Calc [20]
		3.066	13.312								Exp [26]
Cr_2_GaC		2.900	12.632								Exp [26]
Sc_2_GaC	LDA-CA-PZ	3.236	14.394		4.44						Calc [45]

(B* is the bulk modulus obtained from the Birch–Murnghan equation of energy of state (EOS)).

**Table 2:** The elastic constants (in GPa) for M_2_GaC (M = Zr and Hf) obtained from GGA and LDA.

M_2_GaC	Functional	C_11_	C_12_	C_13_	C_33_	C_44_	B_v_	B_r_	G_v_	G_r_
Zr_2_GaC										
	PBE	266.78	59.35	59.91	217.99	91.07	123.32	122.33	95.33	94.66
	PW91	281.03	67.30	76.23	218.78	81.24	135.60	134.28	91.27	89.39
	LDA	296.52	70.35	75.63	244.41	94.46	142.30	141.42	101.45	100.46
Hf_2_GaC										
	PBE	305.81	65.32	72.60	251.48	112.85	142.68	141.84	112.69	111.87
	PW91	305.71	66.90	73.44	249.97	111.07	143.21	142.28	111.49	110.66
	LDA	340.36	75.95	88.05	284.29	126.99	163.23	162.55	124.77	123.76

**Table 3:** The calculated bulk modulus (B), shear modulus (G), Young’s modulus (E), Pugh’s ratio (B/G and G/B), anisotropic index (A), and Poisson’s ratio for M_2_GaC MAX phase (M = Zr, Hf).

M_2_GaC	Functional	B (GPa)	G (GPa)	E (GPa)	B/G	G/B	A	υ
Zr_2_GaC								
	Ref [34]	146						
	PBE	122.827	94.99	226.57	1.29	0.77	0.99	0.192
	PW91	134.938	90.33	221.56	1.49	0.67	0.94	0.226
	LDA	141.857	100.96	244.80	1.40	0.71	0.97	0.212
Hf_2_GaC								
	Ref [34]	158						
	PBE	142.260	112.28	266.68	1.26	0.79	1.09	0.187
	PW91	142.749	111.06	264.58	1.28	0.77	1.08	0.191
	LDA	162.893	124.27	297.21	1.31	0.76	1.13	0.195

**Table 4:** The calculated transverse elastic wave velocity (*v_t_*), longitudinal elastic wave velocity (v_l_), the average wave velocity (*v_m_*), Debye temperature (*θ_D_*), thermal expansion coefficient (α), and heat capacities at constant volume and constant pressure (Cv and Cp) at 300 K for M_2_GaC MAX phase (M = Zr, Hf).

M_2_GaC	*v_t_* (×10^3^ m/s)	*v_l_* (×10^3^ m/s)	*v_m_* (×10^3^ m/s)	$\theta_{D}$ (K)	α (×10^−5^ K^−1^)	C_V_ (J/mol·K)	C_P_ (J/mol·K)
Zr_2_GaC	3.85	6.24	4.24	787.91	1.1432	72.33	72.77
Hf_2_GaC	3.21	5.18	3.54	720.78	0.8427	76.00	76.34
